# Weather Effects on the Patterns of People's Everyday Activities: A Study Using GPS Traces of Mobile Phone Users

**DOI:** 10.1371/journal.pone.0081153

**Published:** 2013-12-18

**Authors:** Teerayut Horanont, Santi Phithakkitnukoon, Tuck W. Leong, Yoshihide Sekimoto, Ryosuke Shibasaki

**Affiliations:** 1 Department of Civil Engineering, School of Engineering, The University of Tokyo, Tokyo, Japan; 2 Computing and Communications Department, The Open University, Milton Keynes, United Kingdom; 3 Faculty of Engineering and IT, University of Technology Sydney, Sydney, Australia; University of Namur, Belgium

## Abstract

This study explores the effects that the weather has on people's everyday activity patterns. Temperature, rainfall, and wind speed were used as weather parameters. People's daily activity patterns were inferred, such as place visited, the time this took place, the duration of the visit, based on the GPS location traces of their mobile phones overlaid upon Yellow Pages information. Our analysis of 31,855 mobile phone users allowed us to infer that people were more likely to stay longer at eateries or food outlets, and (to a lesser degree) at retail or shopping areas when the weather is very cold or when conditions are calm (non-windy). When compared to people's regular activity patterns, certain weather conditions affected people's movements and activities noticeably at different times of the day. On cold days, people's activities were found to be more diverse especially after 10AM, showing greatest variations between 2PM and 6PM. A similar trend is observed between 10AM and midnight on rainy days, with people's activities found to be most diverse on days with heaviest rainfalls or on days when the wind speed was stronger than 4 km/h, especially between 10AM–1AM. Finally, we observed that different geographical areas of a large metropolis were impacted differently by the weather. Using data of urban infrastructure to characterize areas, we found strong correlations between weather conditions upon people's accessibility to trains. This study sheds new light on the influence of weather conditions on human behavior, in particular the choice of daily activities and how mobile phone data can be used to investigate the influence of environmental factors on urban dynamics.

## Introduction

People habitually carry their mobile phones with them much of the time as this pervasive technology offers its users a means for constant and available communication as well as personal entertainment. However, the accompanying mobile phone can also provide researchers with an efficient tool for capturing human mobility pattern. Through this, researchers have a unique opportunity to get a better understanding of the individual as well as social behaviors that collectively shape our society. Along with the logs of incoming and outgoing calls, telecom operators can also capture people's phones movement, as the phone move through the ubiquitous network of towers. This transforms the phone into individual life loggers, giving longitudinal records of personal mobility while offering unprecedented fine-grained data at the aggregate level. This can give researchers a glimpse of various dimensions of human life. For example using mobile phones to study social structure [1], how an individual's diversity of social network can lead to greater personal economic development [2], and how weather affects people's use of phone calls to connect with others [3].

Various researchers have used location traces of connected cellular towers of mobile phones to study human mobility, which is important for urban planning and traffic engineering (e.g., [4] [5] [6] [7] [8] [9] [10] [11]). Several aspects of human mobility have been explored and described. For example, human trajectories show a high degree of temporal and spatial regularity with a significant likelihood of returning to a few highly visited locations [4]. Trajectories of human mobility follow the principle of exploration and preferential return, which governs the way people explore new places while often returning to the previously visited locations [5]. Others try to predict individual mobility by examining phone location traces data (i.e., phone movement) in conjunction with datasets containing geographical features such as point of interest (POI) and land-use information [6]. Despite the differences in people's travel patterns, there is a strong regularity in their mobility on a regular basis, which makes 93% of people's whereabouts predictable [7]. Developing an understanding of mobility patterns (through phone location trace data) has helped with detecting the outbreak of mobile phone viruses [8], comparing people flow between cities [9], identifying commuting patterns [10], and understand the geography of social networks [11].

These emergent studies of mobility have mainly focused upon modeling, predicting, and analyzing human mobility data between cities. However, these approaches often miss out on the richer context of mobility, such as the type of activities that people might be engaged with at the locations they travel to. After all, people move between places in the city for different purposes. Besides travelling between home and work, they are also engaged in activities related to the place they visit, for example, eating in a restaurant, shopping or browsing in a mall, and jogging in a park. Thus, developing methods to help us infer the types of activities associated with different public places can offer a richer characterization of people's daily activity patterns, which has many potential benefits, such as facilitating urban design and management. In this paper, we describe an approach to characterize human daily activity patterns using detailed location traces of mobile phones and spatial profiles. To build upon, and extend a previous investigation that demonstrates how weather conditions could impact people's mobile social interactions, this investigation shows how we can glean further insights into people's behavior with regards to their daily activities by looking for correlations with detailed information about weather conditions. After all, research has shown that weather can affect people's behaviors such as their mood [12] [13] thermal comfort level [14] [15], and social interaction [3]. The weather can also influence traffic demands, and how we travel [16] [17] [18], public health [19], crime rates [20], and even stock prices [21] [22]. Thus, this paper describes our investigation into how weather shapes people's patterns of mobility and the associated activities in the Tokyo metropolis of Japan. We will refer to this area as Tokyo for the rest of the paper.

## Methodology

This section will describe the datasets used and the analysis carried out in this study. Given that the cities we live in are increasingly associated with unprecedented amount of data that is being produced and capture, we will demonstrate how we analyze some of these datasets to reveal correlations and hidden patterns of inhabitants in a large metropolis such as Tokyo. Through this, we hope to produce knowledge that can inform better urban planning in ways that are responsive to the needs of its inhabitants.

### Datasets

We used three datasets in this study. The first dataset is the GPS location traces of mobile phone users in Tokyo. The data was collected for a full calendar year from 1st August 2010 to 31st July 2011 during which the location of each mobile phone user was recorded continuously. To reduce battery consumption, the accelerometer was used to detect periods of relative stasis during which power-consuming GPS acquisition functions can be suspended. The sampling rate thus varied with the user's mobility. However, the rate of sampling did not exceed once every five minutes.

**Figure 1:**
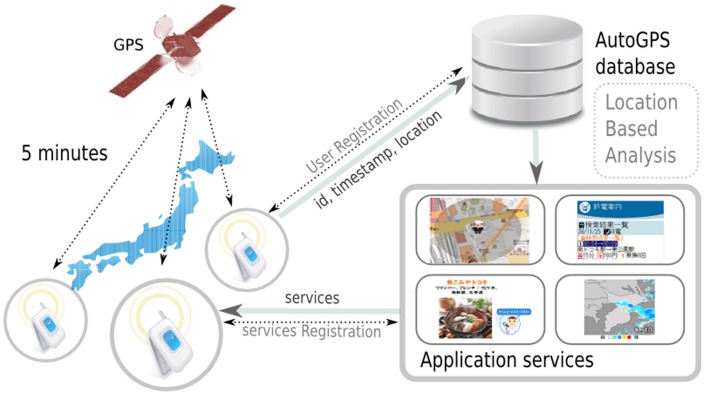
Overview of data collection process.

**Figure 2:**
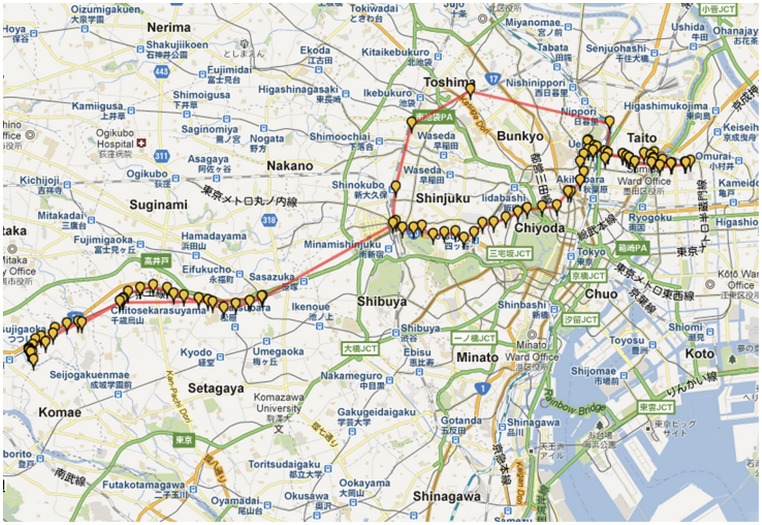
An example of a mobile phone user's location traces.

A leading mobile phone operator in Japan provided this mobile phone GPS dataset. In particular, the dataset was derived from mobile phone users who registered for location-based services. The location information was sent through the network and used to perform specific analysis from which certain services were then provided for the registered users, as shown in Fig. 1. As part of this service, the mobile phone users were aware that their locations were being recorded. Furthermore, to preserve user's privacy, the dataset was completely anonymized by the company. Each entry in the dataset included: unique user ID, position (latitude, longitude), timestamp, altitude, and approximated error (i.e., <100 m, <200 m, or <300 m). This dataset provided finer grained location traces than regular mobile phone call detail records (CDRs) in which the user's location is recorded only when the connection to cellular network is established e.g., making/receiving a call and sending/receiving text message. As an example, Fig. 2 shows location traces of a mobile phone user.

**Figure 3:**
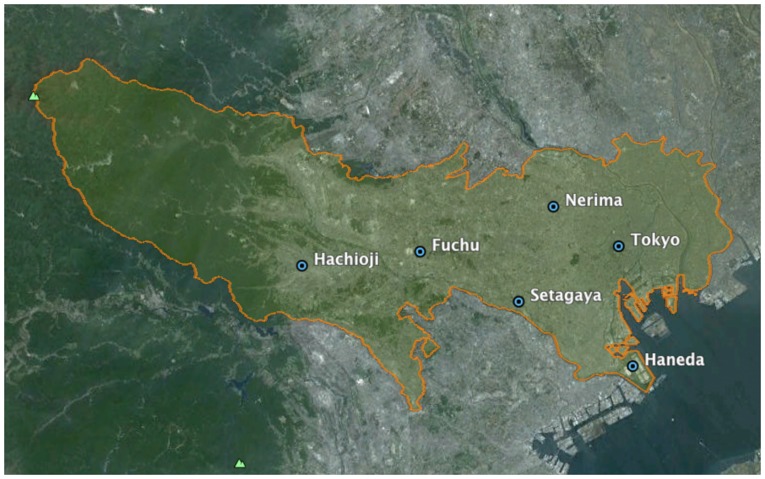
Locations of weather stations from which the data was gathered for the study. The area considered in this study is enclosed by highlighted contour line.

**Figure 4:**
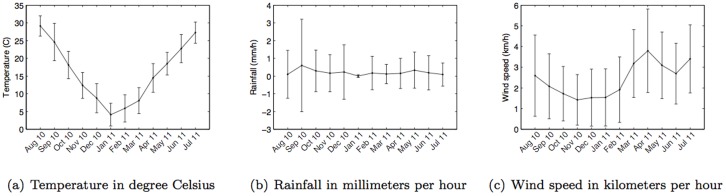
Monthly weather conditions in Tokyo during the period of the analysis.

The second dataset is the weather conditions of Tokyo. This information was collected from Metbroker [23]. MetBroker currently provides access to twelve databases of information collected from seven different countries. It is mainly used to supply important input for agricultural models but the information is also useful for our investigation. MetBroker is a legacy weather database, which provides seamless integration of sensor network from different weather stations with a standard format. Hence, it is a reliable data source used by researchers. In this study, hourly information of the *temperature* (degree Celsius), *rainfall* (millimeter per hour), and *wind speed* (kilometers per hour) of Tokyo from 1st August 2010 to 31st July 2011 were gathered from six different weather stations – their geographical locations are shown in Fig. 3. Monthly statistical means and standard deviations of each weather parameter during the time of the analysis are shown in Fig. 4.

**Figure 5:**
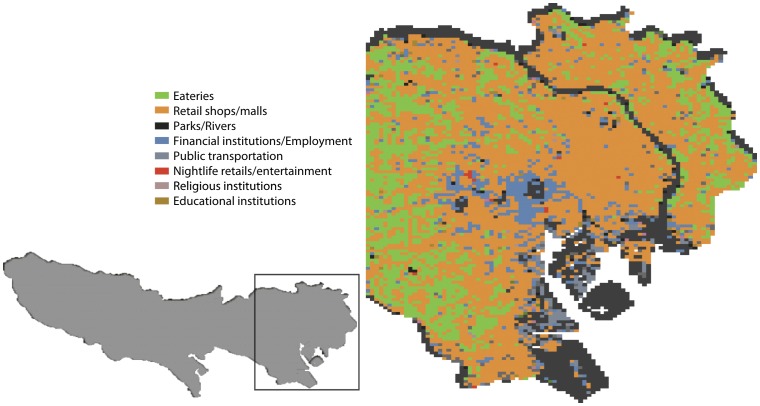
Partial view of Tokyo map in which most probable activities were inferred according to the yellow-pages information for each grid cell area.

The third dataset used in this study is the national phone directory (Yellow Pages) data collected from Telepoint [24]. About 28 million addresses nationwide are geocoded (latitude, longitude) and the information is updated every two months. The data from October 2010 was chosen because this was the most recent update of the database for the period chosen for this study. These addresses were grouped into 14 categories. However, we only used eight of the categories that are associated with activities that people engage with within these 52 municipalities. The other categories were related to residential categories, empty spaces, agricultural fields, etc. This was achieved using the same approach we had described in our previous work to construct the *Activity-Aware Map* [25]. This involves categorizing each 250 m-by-250 m grid cell using the Weight-Area method, i.e., each cell is assigned the most probable activity, i.e., the most dominant activity in the cell based on space profile category. The (most popular) space profile categories and their corresponding activities are shown in Table 1. Based on the Yellow Pages information, a map that presents most probable activities in different cell areas were constructed. A partial view of Tokyo's activity map can be seen in Fig. 5 where activities are in different colors.

**Table 1:** Space profiles categories and corresponding activities.

Space profile category	Examples of inferred activities
Eateries	Consuming food and/or beverages
Retail shops/malls	Window shopping, shopping, leisure browsing
Parks/Rivers	Leisure
Financial institutions/Employment	Financial transactions
Public transportation	Catching trains or buses
Nightlife retail/entertainment	Pubs/bars activities or nightlife entertainment
Religious institutions	Religious related activities
Educational institutions	Education related activities

### Analysis

A mobile phone has become a necessity of the modern era and an integral part of our everyday lives. This study takes the advantage of its pervasive use to capture people's mobility and their daily activity patterns. With the detailed location traces of mobile phone users, the mobility pattern of each mobile phone user can be extracted i.e., trajectories of movements and prolonged stops. When people are not commuting, and are at stops, they are most likely involved in some activities. Thus besides being at home, or at work, they could be eating at a restaurant, shopping in mall, sitting in a park, and so on. Therefore in our analysis we assumed that an activity (other than those carried out at home or at work) was engaged only during a ‘stop’. To segment these traces into individual trajectories so that daily mobility pattern of each individual can be identified, we describe here our basic algorithms to extract *trips* and *stops*.

Let *X* represents a set of sequential traces of the user such that 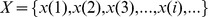 where *x*(*i*) is the *i^th^* location of the user. A stop can be identified as a series of locations in which the user stays in a certain area for a sufficiently long period of time. As each position *i* contains location information (*lat*, *long*) and timestamp (*t*) i.e., 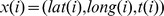, a stop is thus regarded as a sequence of positions 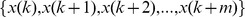 where the distance between any any positions is less than the spatial threshold *S_th_* i.e., 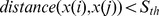 for 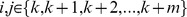, and time spent within the location is greater than the time threshold *T_th_* i.e., 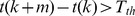. The position *x*(*k*) thus becomes the last position of the previous trip while *x*(*k*+*m*) becomes the first position of the next trip.

**Figure 6:**
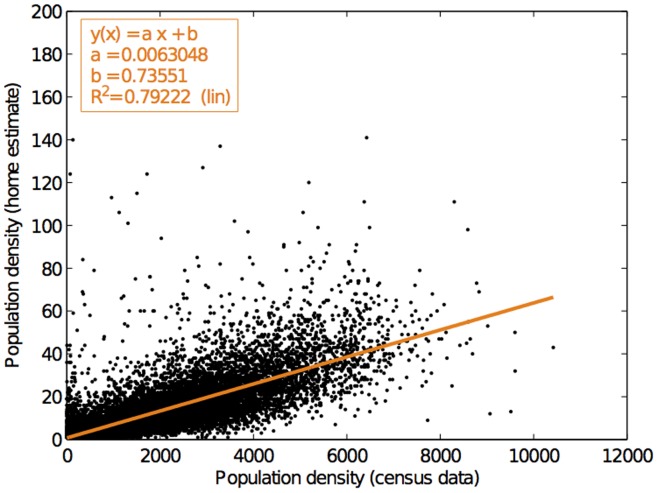
Comparison between our inferred residential municipality population of the mobile phone user subjects and the actual municipality population obtained from census data.

Once the ‘stops’ have been identified, the *home* and *work* locations of each user can then be estimated as the locations of the most frequent stop during the night (10pm–6am) and day hours (9am–5pm), respectively. This estimation approach was found to be fairly reasonable as the result of the estimated home locations was comparable (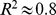) with the area population density information of the 2006 census data [26] (as shown in Fig. 6), and the average computed commuting distance of 24.34 km based on the estimated home and work locations was reasonably close to 26 km of the average commuting distance according to the census data [26]. Based on the above algorithm, we were able to gather 31,855 subjects (from the dataset provided to us) whose home and workplace were within the Tokyo metropolis.

**Figure 7:**
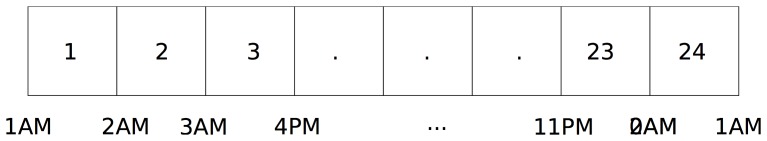
Hourly time frame was used to capture daily activity patterns.

Identification of stops, in addition to home, and work locations for each subject then allowed us to infer people's daily activity pattern, which was defined as a series of most probable engaged activity throughout a day. The daily activity pattern for each subject was constructed using a similar approach to our previous study using cell tower-level location traces of mobile phone users [25]. However, with a higher level of granularity of the data in this study, we were able to better identify the most probable activity for each hour (as shown in Fig. 7). Instead of using three-hour time windows as in [25], we were able to interrogate the data for every hour of the day, to infer the predominant activity during the hour according to the series of stops and categories of visited places for each subject.

Using inferred daily activity patterns, we then further investigated the influence of the weather on people's mobility and their daily activity patterns. We used various weather parameters such as *temperature*, *rainfall*, and *wind speed*. Our data analysis explored the influence of each of these weather parameters upon people's *mobility* in terms of duration of stops and *daily activity patterns* over space and time.

## Results

Although certain weather parameters depend upon others, we considered the effects of each weather parameter separately on people's mobility and activity patterns in this study. The following results show how different weather parameters correlate to people's mobility and variation in activity patterns over space and time.

### Weather effects on mobility and stop durations

People travel for many different reasons. It can be for regular purposeful activities such as to go to work, or to shop, and it could also involve leisurely activities, such as to dine out, to socialize, or for vacation. Understanding the collective mobilities that make up the urban dynamics is important for particular city authorities as it can better inform urban planning and logistics for transportation.

Given that the weather has been found to have significant impact on a number of phenomena related to human behavior, we first wanted to investigate how the weather impacts people's mobility. Therefore we examined the statistical distribution of *stop duration* for different weather parameters. To allow us to more easily discern any emergent patterns we divided each weather-related parameter into a set of ranges, or bands. Based on the climate history of Tokyo (Fig. 4), temperature was considered between −5°C and 35°C, divided into four bands, each with a 10-degree span (−5°C to 5°C, 5°C to 15°C, 15°C to 25°C, 25°C to 35°C) Rainfall was divided into four bands: no rain (rainfall  = 0 mm) and the rest was evenly divided into three bands in the range from 0 mm to 15 mm (0–5 mm, 5 mm–10 mm, 10 mm–15 mm); windspeed in four bands: 0–2 kmph, 2–4 kmph, 4–6 kmph, and stronger than 6 kmph.

We would like to also note that although other researchers have often used the number of visited locations to characterize human mobility (e.g., [4] [5] [7] [11]), it would not be an appropriate approach in this study given that we wanted to compare people's mobility across different bands of weather for each weather parameter and in this investigation, each band has different length of time observation.

**Figure 8:**
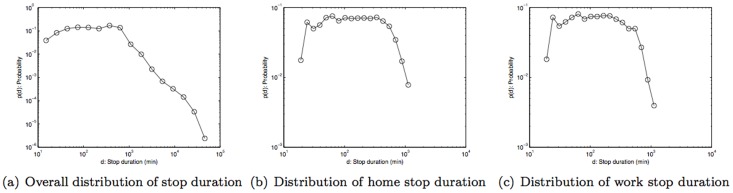
Probability mass function of stop duration of subjects.

The statistical distribution of the stop duration for each band of each weather parameter was computed as a *probability mass function* (*pmf*). This pmf is basically a normalized histogram on the logarithmic scale, where normalization allows comparisons across different bands of each weather parameter, and a logarithmic scale was used because of the nature of the statistical distribution of the stop duration found in this study as shown in Fig. 8(a). In addition, pmf of stop duration for home and workplace are shown in Fig. 8(b) and 8(c) respectively.

**Figure 9:**
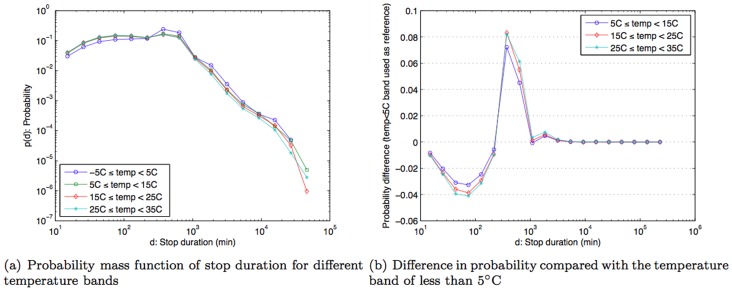
Probability mass function of stop duration under different bands of temperature and probability difference compared with the temperature band of less than 5°C.

**Figure 10:**
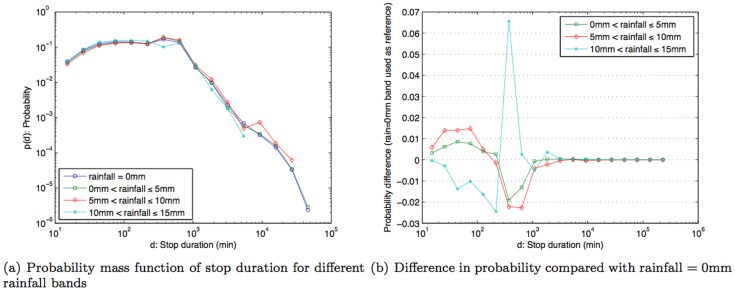
Probability mass function of stop duration under different bands of rainfall and probability difference compared with the band of rainfall  = 0 mm.

**Figure 11:**
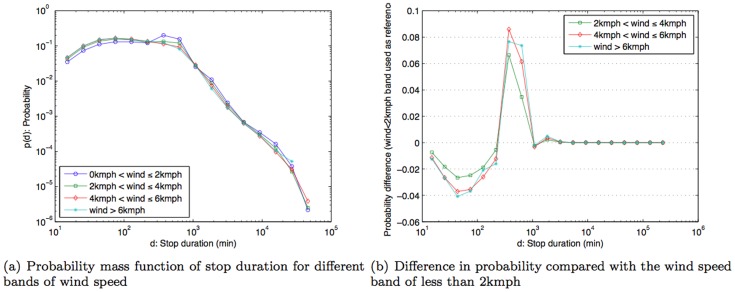
Probability mass function of stop duration under different bands of wind speed and probability difference compared with the wind speed band of less than 2

The results in Fig. 9 show that the pmf for the temperature band −5°C to 5°C is higher than other bands when the stop duration that is two hours or more. The result is statistically significant with *p*-value  = 3.0616×10^−4^, based on Fisher's exact test with the total number of stops in very cold weather (temperature <5°C)  = 262,803, number of stops in very cold weather that are longer than two hours  = 188,905, number of stops in other temperature bands  = 2,757,720, and number of stops in other temperature bands that are longer than two hours  = 1,705,941. In other words, when compared to other temperature bands, there is a noticeably higher likelihood that people make stops that are two hours or longer on very cold days. Conversely, for days with weather above 5°C, people are more likely to make stop durations that are less than two hours. As guidance on variability, Fig. 9(b) shows the difference in probability measures when comparing other temperature bands against (−5°C to 5°C)-band i.e., subtracting probability measure of other bands from (−5°C to 5°C)-band. Hence, positive difference implies that (−5°C to 5°C)-band has a higher probability and vice versa. Rainfall, on the other hand, does not appear to influence how long people choose to stop (Fig. 10). On the other hand, wind speed (similar to temperature), exhibits a correlation to stop duration. There is a higher likelihood (higher pmf) of stop duration of two hours or more for relatively calm days where wind speed is between 0–2 kmph (Fig. 11). Statistical significance test yields *p*-value  = 1.829×10^−9^ with the total number of stops in calm weather (wind speed <2 kmph)  = 1,855,467, number of stops in calm weather that are longer than two hours  = 1,230,570, number of stops in other wind-speed bands  = 1,489,735, and number of stops in other wind-speed bands that are longer than two hours  = 867,471. Thus, besides work and home, people don't often make stops of about two hours or longer. The exceptions are on very cold days or on relatively calm days.

Furthermore, through the Activity-Aware Map approach of using Yellow Pages information, we set out to infer the activities for stops that people made which were two hours or longer. By discounting locations identified as home and workplace, and using the categories we computed in Table 1, we found that people spent about 80% of their longer stops at areas that predominantly consist of eateries or food outlets such as restaurants, cafès, and so on, and 17% at areas that predominantly consist of retailing, such as shops, shopping mall, and so on when the temperature was between −5°C and 5°C. Likewise, when the wind was less than 2 km/h it was observed that people spent about 88% of their longer stops at areas of eateries and food outlets, and 11% in areas of retail and shopping. These results suggest that in a cold or calm (not windy) day, people tend to take their time having meals, snacks, and/or beverages, and to a lesser extent, spending time on shopping-related activities in areas of retail or in a shopping mall, perhaps buying things or simply window shopping.

### Weather effects on activities at different times of the day

Human mobility is periodic and hence highly predictable [4]. Our daily activities are often pre-scheduled as most of us are in the work-life routines. With the daily routine such as going to work in the morning, having lunch around noon, shopping in the afternoon, and going to a pub in the evening that most of us struggle to break, the question is: *does the weather have any impact on such a daily activity patterns*? To find out if the weather affects our daily activities and to what extent, we examined the weather impact across different hours of the day. The *entropy* of activities was used to capture the variation in activities engaged by the subjects. In information theory, entropy (*H*(*X*)) is a measure of uncertainty or randomness associated with a random variable and it can be computed as follows [27]: 
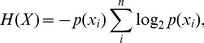
(1)where *X* is a random variable with *n* outcomes 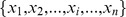 and *p*(*x_i_*) is a probability of outcome *x_i_*.

Entropy value was computed for each hour of the day where *X* represents a set of activities engaged by each of *n* subjects in that particular hour i.e., variables *x_i_* presents activity of the subject *i*. The probability *p*(*x_i_*) was then computed as a ratio of the number of times the activity *x_i_* was observed in the hour across all *n* subjects to the total number of subjects (which is *n* = 31,855 in our case). The entropy was chosen in this investigation because it was suitable for categorical variables, which represented activities in this study. A higher entropy value implies a higher randomness in activities among the subjects. Entropy equals to zero means no randomness i.e., all activities engaged by subjects are the same.

**Figure 12:**
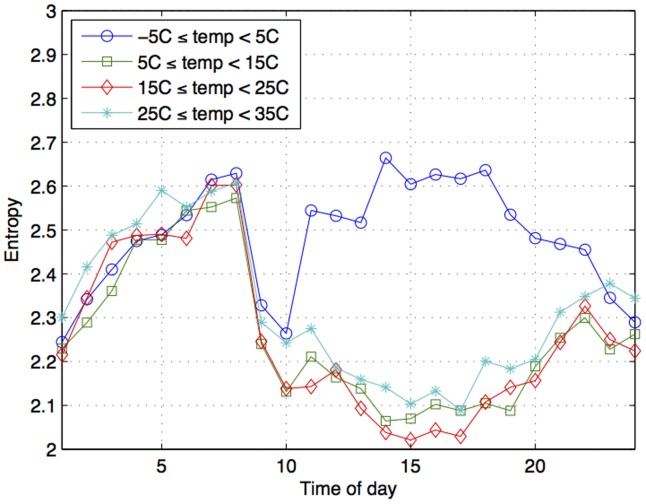
Entropy values across time of the day under different bands of temperature.

With the activity pattern inferred in the same way as described earlier for each band of each weather parameter, the results show that different weather conditions do have an influence on people's activity patterns throughout the day. As Fig. 12 illustrates, regardless of the day's temperature range, entropy values show a dramatic decrease between 8AM and 9AM, where there is generally low variation in people's activities. Given the timeframe, this may likely be due to the fact that most people are traveling – on their way to work. After 10AM, the effect of different temperature bands becomes more distinct. The effect of the temperature band −5°C to 5°C stands apart, and in opposition to the other temperature bands. For this band, entropy values increases from 10AM reaching the highest between 2PM and 6PM before decreasing (see Fig. 12). By 10PM the entropy value for this band is still higher than that of the other bands. In other words, on very cold days (−5°C to 5°C), people's activities are found to be most varied from 10AM onwards.

For the other temperature bands, (temperature bands above 5°C), the opposite effect is observed. The least varied activities are observed for the temperature band of 15°C and 25°C, especially between 12PM and 5PM. In comparison, the temperature band of 5°C to 15°C shows slightly higher level of entropy values, followed by the temperature band between 25°C and 35°C. In other words, for the three temperature bands that are above 5°C, people's activities show the least variations within the relatively comfortable temperature conditions of 15°C to 25°C, while in the highest temperature band of 25°C to 35°C, people show more variations in their activities. In fact, on days when the temperature is 5°C, people's activities begin to increase in variation after 5PM well into the night. Of these, the warmest days (25°C to 35°C) appears to show the highest variation in activities at night. In other words, people tend to engage in a wide range of different activities on very warm nights.

**Figure 13:**
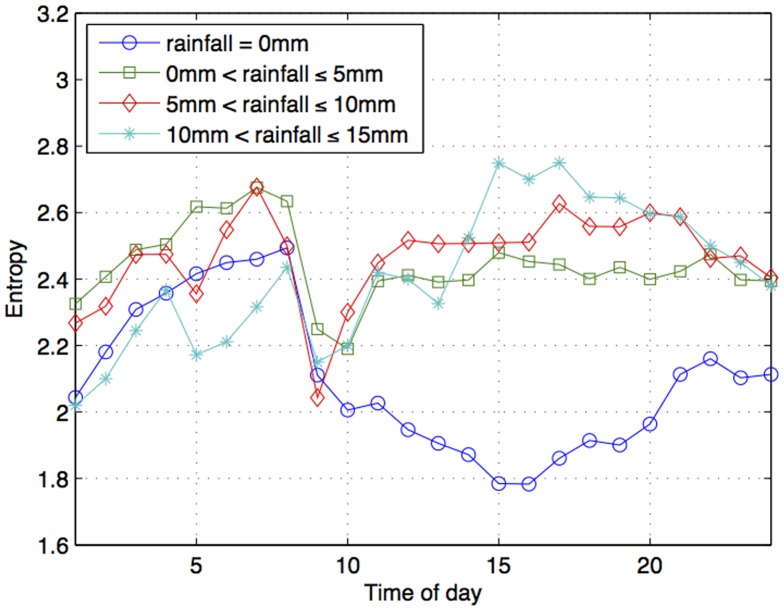
Entropy values across time of the day under different bands of rainfall.

If very cold days lead to high variations in people's activities, rainfall has a similar effect. In fact, the heaviest rainfall band (10 mm–15 mm) shows, an entropy level that is even higher than that of a very cold day. In other words, people's activities are the most varied on days with the heaviest rainfall. Again, just like that for temperature, entropy values show a dramatic decrease between 8AM and 9AM, regardless of the amount of rainfall. This picture changes after 9AM (Fig. 13). One band: no rainfall (rainfall  = 0 mm), stands apart from the rest. People's activities decreases in entropy values (i.e., decreases in variation of activities) on dry days, from 9AM onwards, reaching the lowest entropy values between 3PM and 4PM.

However, rainfall (when rain is >0 mm) shows an opposite trend to dry days. On days that receive rainfall we observe an increase in entropy values from 9AM, i.e., increase in variation of people's activities. In fact, the wetter the day (i.e., the higher the rainfall), the more varied people's activities tend to be. For example, in the highest rainfall band (10 mm to15 mm), we observe that activities tend to be the most varied, especially between 3PM–7PM. Lower rainfall also lead to varied activities although not as much over the day.

**Figure 14:**
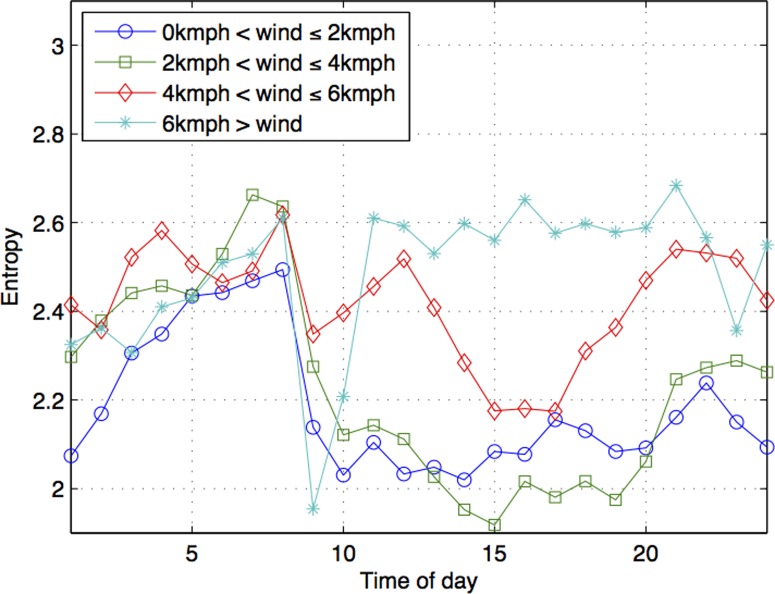
Entropy values across time of the day under different bands of windspeed.

The effect of wind is slightly more different during the day from that of temperature and rainfall. In fact, we observer that wind speed has the most varied effect on people's activities. When wind speed is above 4 kmph, we generally see higher entropy values (Fig. 14). This is most obvious with the highest wind speed band (when it is 6 kmph or greater). This has a profound effect on the entropy values, especially from 11AM10PM. On days with such high wind speed, people activities are highly varied, especially within that time frame. On the other hand, on days with the highest wind speed band, there is a dramatic drop in entropy values between 8AM and 9AM. Thus, on high wind mornings, there is a very low variation in people's range of activities. However, relatively calm days (0 kmph to 2 kmph) do not consistently show lower entropy values. Between 1PM and 8PM it is days with wind speed between 2 kmph and 4 kmph that shows the lowest entropy values. In other words, on days with slight winds, people's activities show the least variations, especially between 1PM and 8PM.

### Weather effects on activities in different areas of the city

The impact of weather upon people's activities, and to what extent it influences people's choice of activities may also depend upon where they live in Tokyo. This is particularly important given the size of Tokyo metropolis. This could involve geographical features of the urban landscape such as shopping malls, hospitals, places of worship, parks and so on. In large metropolises, it can also involve public transport networks.

To observe how the weather impacts on different areas of Tokyo, the entropy was again used as a measure of the variation in activity patterns. This time, we computed the weather's impact as a difference in entropy values between the regular and irregular weather conditions for each of Tokyo's 52 municipalities. Based on the results from the previous section on temporal weather effects, we defined the regular condition for each weather parameter as 5°C to 35°C for temperature, 0 mm for rainfall, and 0 to 4 kmph for wind speed. The irregular condition for each weather parameter is thus −5°C to 5°C, >0 mm, and >4 kmph for temperature, rainfall, and wind speed, respectively.

**Figure 15:**
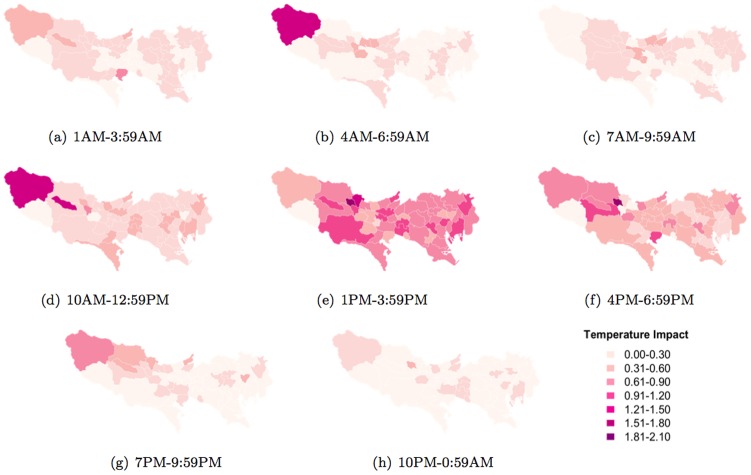
Temperature impact (change in entropy between normal and abnormal temperatures) on daily activity patterns across different municipalities.

**Figure 16:**
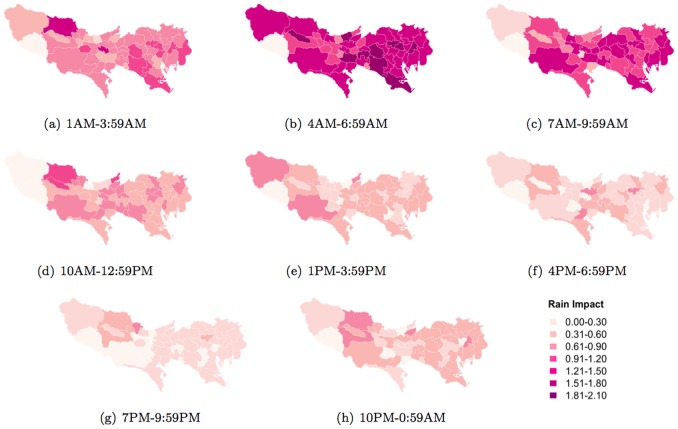
Rain impact (change in entropy between normal and abnormal temperatures) on daily activity patterns across different municipalities.

**Figure 17:**
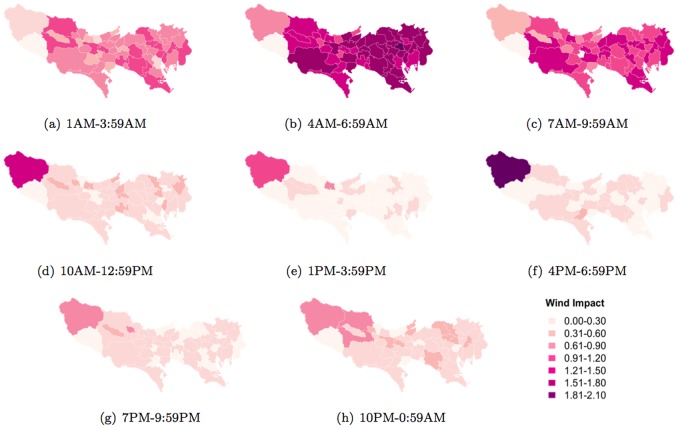
Wind impact (change in entropy between normal and abnormal temperatures) on daily activity patterns across different municipalities.

The results, which can be seen in Fig. 15, 16, and 17, show that each weather parameter has varying impact across different parts of Tokyo at different times of the day. Figure 15 shows how temperature impacts people's normal activity pattern in different parts of Tokyo. In addition, the impact varies for different times of the day. For example, we can see in Fig. 15(b) that weather impacts significantly upon people living in the western region of Tokyo between 4AM and 6.59AM, and also between 10AM and 12.59PM, when compared to other regions. However between 1PM–3.59PM, the impact is more diffused across all of Tokyo.

The impact of rain on people's normal activities is most discernible across all areas of Tokyo between 4AM–6.59AM (Fig. 16(b)) and gradually decreasing in impact between 7AM–9.59AM (Fig. 16(c)). Windy days show particularly interesting patterns, again, especially in the western region of Tokyo (Fig. 17)). Firstly, while wind generally impact people's normal activities in most areas of Tokyo, especially between 4AM–9.59AM, this impact is not as significant in the western region. However the impact of wind becomes more prominent (significantly more profound than other areas of Tokyo) from 10AM onwards peaking between 4PM–6.59PM.

The varying impacts described above could be due to a number of influential factors. One of them is the ability for people to move around under different weather conditions. Therefore, we chose to further investigate these findings in light of people's accessibility to public transportation. For each municipality, we computed the distance between the subject's home location and the nearest public transport hub as a measure of the accessibility of public transport. As buses and trains are the main public transports in Tokyo, bus stops and train stations were thus chosen as public transport hubs in this investigation.

It has been shown that people's activities in Tokyo are strongly shaped by their ability to access trains [28]. However, in some areas of Tokyo, the distribution of train stations may not be as dense, people are further away from available train stations. In fact, there is one town in the furthest west that has only one train station with no one in our study population living within 2 km of that train station. So we wanted to see if people's proximity to a train station affects their choice of activities under different weather parameters.

**Figure 18:**
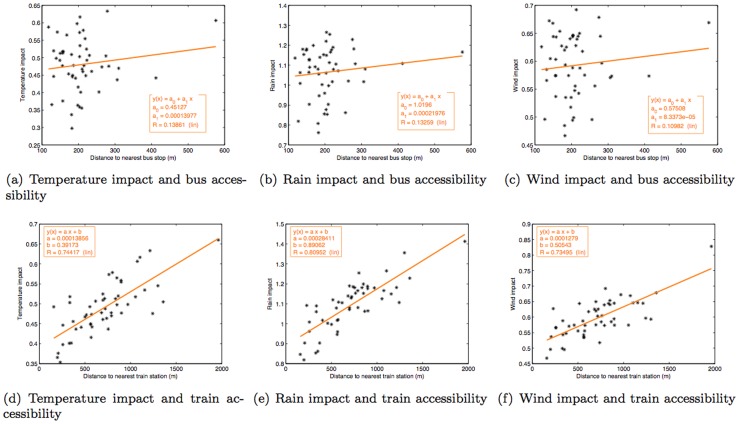
Relationship between the weather impact and accessibility of pubic transportation.

The results (Fig. 18) show that the further away the person is from a train station, the bigger the effects that particular weather pattern has on people's choice of activities (R^2^ = 0.7∼0.8). On the other hand, people are never far from bus stops (average distance  = 215 m). Thus, in terms of buses, people's proximity to bus stops appears to show that the varying weather patterns do not discernibly affect people's choice of activities.

We also performed the same analysis for people's proximity to other elements of urban infrastructure (i.e., hospitals, shopping malls, parks, and night clubs) but did not observe strong correlations. The R^2^ values are shown in Table 2.

**Table 2:** The R^2^ values representing the correlation between the impact of different weather parameters and accessibility of urban infrastructure.

Urban infrastructure	Weather parameters		
	Temperature	Rain	Wind
Train station	0.744	0.810	0.735
Bus stop	0.451	0.133	0.110
Hospital	0.223	0.070	0.051
Shopping mall	0.073	0.194	0.142
Park	0.376	0.080	0.215
Night club	0.087	0.055	0.025

## Conclusions

The growing availability of big data offers significant potential for researchers to better interpret human behavior and gain insights into various domains. In this study, we analyzed detailed location trajectories of 31,855 mobile phone users in Tokyo for one full calendar year. Daily activity patterns of mobile phone users were estimated based on their location traces and the yellow-pages information. We were particularly interested in the effect of the weather that has on people's daily activity patterns in this study where temperature, rainfall, and wind speed were considered as weather parameters.

While we found interesting variations on how different weather parameters affects people's mobility and activities in Tokyo, we will summarize the most significant findings.

On days that are very cold (−5°C to 5°C), or calm (i.e., wind speed is less than 2 km/h), we found that people were more likely to stay longer and spend more time at areas that consists of eateries and food outlets such as restaurants, cafès, and so on, and (to a lesser degree) at shopping areas with retail outlets, shopping malls, and so on. Moreover, on very cold days, we found that people's activities were more diverse during the daytime, especially after 10AM, and showing greatest variations between 2PM and 6PM. Similarly, we found that people's activities were more diverse on rainy days (especially between 10AM-midnight) as well as on days when the wind speed was stronger 10AM–1AM when wind speed was stronger than 4 km/h (mean  = 2.6 km/h). Finally, we observed different weather impacts for different geographical areas. It appears that the furthest western region of Tokyo shows the most distinct interruption by very cold weather, significantly disrupting its inhabitants' normal activities in the early mornings and before mid-day. By characterizing various areas by people's accessibility to urban infrastructure (i.e., distance to the nearest accessible point), we found strong correlations between the impact of weather and the local inhabitants' accessibility to train stations.

There are nevertheless some limitations to the observations we present in this study. There could be slightly differences in weather conditions that people experienced in areas that were not near the weather stations considered in this study, which may play a role in the findings. How people actually feel about the weather is also subjective and remains an open question, especially when dealing with the effects of weather on such a high level and at such a large-scale. Participatory mobile sensing (e.g., [29]) perhaps is one of the promising approaches. Although there is an interdependency among weather parameters and the way that people experience the overall weather condition, we did not consider this complexity in the current study. Future studies can take this into consideration in order to obtain a more detailed understanding of the overall effect of the weather. The effect of important social events such as New Year and Christmas or emergency events such as earthquake were not considered in the current study, which could possibly have some influence on the findings. This too remains to be explored in our future studies. Finally, due to sparseness of GPS data and resolution of the spatial profile characterization used in this study, the activity patterns inferred based on location traces and Yellow-Pages information may not represent the actual activities in reality. Nonetheless, we believe that the findings of this study to a large extent represent a new knowledge about the influence of the weather that it has on our behavior, in particular the patterns of our physical activities.

Having the ability to combine large datasets to discern particular patterns in human behavior in large metropolises will become more important as the global trend towards urbanization continues. With more people moving to cities, and as cities grow; the capacity to be able to provide a whole range of services, effectively and efficiently, as well as maintaining the safety of its inhabitants is just one of the major challenges facing urban planners. This paper provides an example of how we can begin to use big data in ways that can provide richer contexts into people's behavior and activities so as to add to our understanding of inhabitants in a big city. By developing ways that can help us infer not only mobility but also where people stop, and what people do (besides being at home and at work) under particular weather conditions, we can imagine how such information can inform a whole range of decisions. On an immediate level, this includes the scheduling of public transportation, the planning of future transport hubs, the staffing levels of retail/restaurants, the opening times of such services, and so on. Knowing where people concentrate in light of weather patterns can also be used to predict how security and emergency services can be best deployed. Of course, such capacity to obtain live models and create predictive models of inhabitants in large metropolises can be greatly improved through greater access to better quality and higher resolution data, and by refining our approaches as well as computational efforts.

This approach also opens up to future efforts whereby researchers can combine different sets of data to help mitigate some of the problems that beset rapidly growing metropolises around the world, such as to help deal with wastes, tackle pollution, and even reduce crime by knowing where police deployment is more useful and likely to be effective. As our findings reveal, we believe that working with Big Data can quickly reveal patterns and trends that offer great inference power to shape people's lives. However, it is through close collaborations with other research disciplines such as urban planning, environmental scientists, and sociologists, that we can truly realize the potential to support and enhance people's lives in big metropolises in positive ways.

### Ethics Statement

This research was granted ethical approval from the IRB at Center for Spatial Information Science (CSIS), the University of Tokyo, Japan. The ethics committee review board includes:

1. Prof. Yasushi Asami
2. Prof. Takashi Oguchi
3. Prof. Ryosuke Shibasaki
4. Prof. Takaaki Takahashi
5. Prof. Masatoshi Arikawa
6. Prof. Kaoru Sezaki

The ethics committee review board waived the need for written informed consent from the participants because the data used in the study was anonymized by another company, on behalf of the network provider prior to releasing this data to the authors. The anonymization ensured that no connection could be made between the data and the individual mobile phone users. The mobile phone users had agreed to the terms and conditions for using the services provided by the network operator in order that their communications may be recorded and analyzed to improve the delivery of services.

### Note

The data used in this study cannot be made publicly available due to individual privacy concerns. However, we can make a sample of data available to other researchers upon request.
